# Atomic force microscopy imaging for nanoscale and microscale assessments of extracellular matrix in intervertebral disc and degeneration

**DOI:** 10.1002/jsp2.1125

**Published:** 2020-09-23

**Authors:** Meagan A. Cauble, Nickolas S. Mancini, Judith Kalinowski, George Lykotrafitis, Isaac L. Moss

**Affiliations:** ^1^ UConn Health Department of Orthopaedic Surgery Musculoskeletal Institute Farmington Connecticut USA; ^2^ Department of Mechanical Engineering University of Connecticut Storrs Connecticut USA

**Keywords:** degeneration, extracellular matrix

## Abstract

Degeneration of the intervertebral disc (IVD) is a condition that is often associated with debilitating back pain. There are no disease‐modifying treatments available to halt the progression of this ubiquitous disorder. This is partly due to a lack of understanding of extracellular matrix (ECM) changes that occur at the micro‐ and nanometer size scales as the disease progresses. Over the past decade, atomic force microscopy (AFM) has been utilized as a tool to investigate the impact of disease on nanoscale structure of ECM in bone, skin, tendon, and dentin. We have expanded this methodology to include the IVD and report the first quantitative analysis of ECM structure at submicron size scales in a murine model for progressive IVD degeneration. Collagen D‐spacing, a metric of nanoscale structure at the fibril level, was observed as a distribution of values with an overall average value of 62.5 ± 2.5 nm. In degenerative discs, the fibril D‐spacing distribution shifted towards higher values in both the annulus fibrosus and nucleus pulposus (NP) (*P* < .05). A novel microstructural feature, *collagen toroids*, defined by a topographical pit enclosed by fibril‐forming matrix was observed in the NP. With degeneration, these microstructures became more numerous and the morphology was altered from circular (aspect ratio 1.0 ± 0.1) to oval (aspect ratio 1.5 ± 0.4), *P* < .005. These analyses provide ECM structural details of the IVD at size scales that have historically been missing in studies of disc degeneration. Knowledge gained from these insights may aid the development of novel disease‐modifying therapeutics.

## Graphical abstract

We have expanded atomic force microscopy (AFM) imaging to the realm of the intervertebral disc. Characterization of a murine model for intervertebral disc degeneration reveals hierarchical changes at macro, micro, and nano size scales.

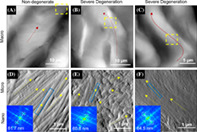

## 1 INTRODUCTION

Degeneration of the intervertebral disc (IVD) is closely linked to the development of low back pain, the leading cause of disability worldwide.^1^ Despite the global impact of this condition, the mechanisms responsible for the development of degenerative disc disease (DDD) are not well understood and treatment is limited to the end‐stage disease with pain management and/or surgery. During degeneration, the IVD tissue undergoes substantial changes to the extracellular matrix (ECM) composition and overall tissue architecture. Understanding fine details in ECM structure and how these changes contribute to a degenerative disc will hasten the development of effective therapeutics.

**FIGURE 1:**
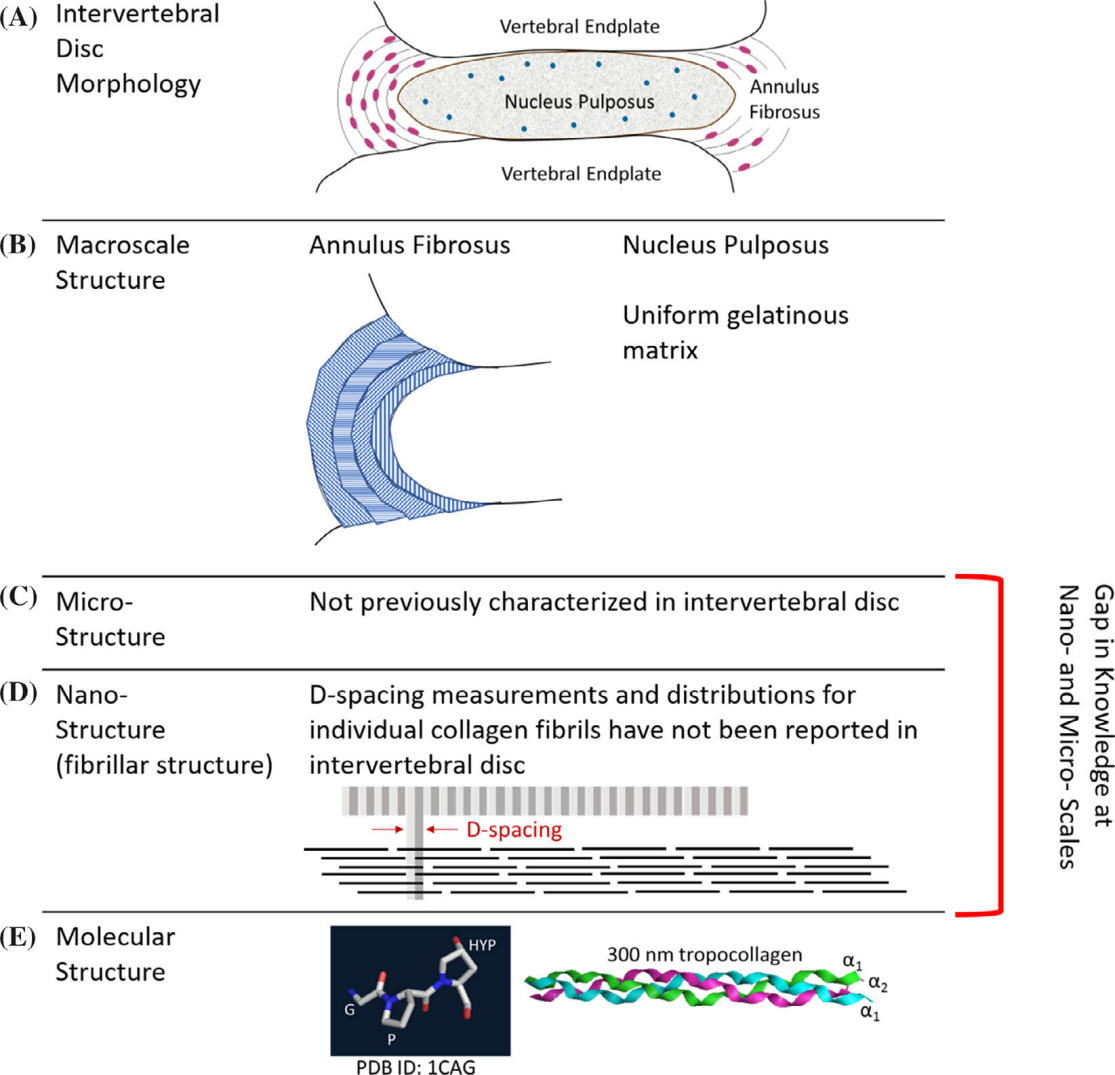
Hierarchical structure of the IVD. A., The IVD is located between vertebral endplates and is composed of the gelatinous nucleus pulposus (NP) and fibrous annulus fibrosis (AF). B, Within the AF lamellae, collagen bundles are organized into parallel bundles that alternate directions in adjacent lamellae. The NP is a uniform gelatinous network of ECM components with no regular macrostructure. C, Microstructure of ECM components is unknown in IVD. D, Nanomorphology of fibril‐forming ECM (type I and type II collagen) has not been measured in IVD. E, Tropocollagen molecules are formed from three collagen alpha peptides: two α1(I) peptide strands and one α2(I) strand for Type I collagen; three α1 strands for Type II collagen. The “Gly‐X‐Y" repeat is essential for triple helix formation. X is commonly proline and Y is commonly hydroxyproline. IVD, intervertebral disc; ECM, extracellular matrix

Collagen, an abundant ECM protein in the disc, plays a critical role in maintaining the structure and function of the IVD and, by extension, the spine.^2^ At the macroscale, the IVD consists of the annulus fibrosus (AF), comprised of type I collagen arranged into concentric lamellae, which surrounds the inner gel‐like nucleus pulposus (NP), comprised of proteoglycan and type II collagen (Figure 1). The abundance of anionic charges from proteoglycans in the NP result in hydration due to osmotic pressure, allowing the disc to resist compressive forces in the spine while the surrounding collagenous AF resists tensile strain. These structure/function relationships are compromised in a degenerative disc. The NP changes to a highly fibrotic tissue with an increased ratio of type I: II collagen. The boundary between the AF and NP becomes blurred and the AF lamellae lose their organized structure and become serpentine.^2^ Meanwhile, ECM structure at the micron and submicron size scales both in healthy disc and the degenerative disc is unknown. The individual collagen fibril organization in the disc at the level of individual fibril orientation, nanomorphology, and microstructure with adjacent fibrils is also not known. This level of submicron ECM structure has been reported for many other musculoskeletal tissues including bone, tendon, dentin, and skin.^3^, ^4^, ^5^, ^6^, ^7^, ^8^ The biological meaning and the molecular origins of collagen D‐spacing have been areas of interest since D‐spacing was first measured. The first widely accepted model for the origin of D‐spacing was proposed in the 1960s by Hodge and Petruska.^9^ Many additional studies have refined this model for the origin of D‐spacing but the problem has remained unsolved. The area has remained an active field of research because the D‐spacing distribution has been shown to be a marker of disease in multiple tissue type, multiple disease states, and multiple species. Although the molecular mechanisms of D‐spacing are yet to be elucidating, investigating these ECM changes still adds to the understanding of musculoskeletal diseases. However, the IVD has been excluded from this area of research. This is likely due to difficulties in sample preparation for AFM with disc and challenges with analysis of such a heterogeneous tissue when the field was just emerging. We report the first imaging‐based AFM study of collagen structure in a murine model for progressive IVD degeneration.

The well‐established murine needle annular puncture model is an easily reproducible method to induce and study IVD degeneration with many studies characterizing the histological, morphological, cellular, and mechanical changes that take place as a result of injury and subsequent degenerative changes.^10^, ^11^, ^12^, ^13^ By 8 weeks, the injured mouse disc is compositionally different from controls with decreased glycosaminoglycan content and increased collagen content.^13^ Additionally, caudal discs in mice with degeneration induced by needle puncture display changes in morphology, cellularity, and composition consistent with those in humans.^10^, ^12^, ^13^ For these reasons, the murine needle‐puncture model was chosen for this study to characterize ECM structure at the nanoscale and microscale. We report alterations to collagen fibril D‐spacing distributions as a function of degeneration and a novel microstructural feature, the *collagen toroid*, which is associated with degeneration in the NP. These findings will begin to fill in the knowledge gap between known molecular structure and tissue macrostructure (Figure 1).

## 2 MATERIALS AND METHODS

### 2.1 Murine model of IVD degeneration

After approval by the institutional animal care committee, surgery was performed on 30 skeletally mature male and female C57BL/6 mice obtained from Jackson Laboratories (Farmington, CT). All mice were aged 10‐13‐weeks‐old, an age consistent with other murine injury models of IVD degeneration, and our surgical procedure was adapted from previously published injury models.^11^ Mice were anesthetized and four caudal discs were exposed via an incision on the dorsum of the tail. Two adjacent discs were injured by puncture of the AF on the dorsal side with a 31‐gauge needle to a depth of 1.75 mm, corresponding to insertion through the NP and partial puncture of the ventral AF. The needle was rotated and then withdrawn. The remaining two exposed discs served as Sham controls. The connective tissue between injured discs was marked with a suture prior to closure of the incision. Buprenorphine was administered at a dose of 0.1 mg per kg of body weight immediately before surgery and twice daily for 3 days following surgery for pain management. The animals were randomized into groups and sacrificed by CO_2_ inhalation at 0 weeks (n = 4), 2 weeks (n = 9), 4 weeks (n = 9), or 6 weeks (n = 8) postsurgery. At time of sacrifice, tails were immediately harvested, the skin removed, and the remaining tissue prepared for evaluation by histology (chemical fixation) or atomic force microscopy (AFM) imaging (cryo‐preservation).

### 2.2 Histology

Tails allocated for histology (0 weeks n = 2, 2 weeks n = 6, 4 weeks n = 6, and 6 weeks n = 4) were chemically fixed in 10% neutral buffered formalin at 4°C for 48 hours. Fixed tissues were cryopreserved by immersion in 30% sucrose overnight at 4°C followed by embedding in Tissue‐Tek optimal cutting temperature (OCT) medium at −80°C. Mid‐sagittal cryosections 10 μm thick were stained with Harris hematoxylin and eosin (H&E) to visualize overall tissue architecture. The Masuda grading system was used by two evaluators, blinded to sample identity, to quantify the grade of degeneration for each stained disc.^14^

PicroSirius Red (PSR) staining was performed on tails allocated to AFM after all imaging was complete (n = 1 per group for PSR staining). 10 μm thick tissue sections were equilibrated in PBS for 5 minutes and then chemically fixed with 4% paraformaldehyde (28 908; Fisher Scientific) in PBS for 10 minutes. From this point, PSR staining was performed as described by others^15^, ^16^ using Direct Red 80 (365 548; Millipore Sigma, Burlington, Massachusetts) dissolved into a saturated aqueous picric acid solution. Samples were viewed under linearly polarized light (Nikon eclipse 50i; Nikon, Edgewood, New York).

### 2.3 Atomic force microscopy

#### 2.3.1 Sample preparation

The mouse tails designated for AFM (0 weeks n = 2, 2 weeks n = 3, 4 weeks n = 3, and 6 weeks n = 4) were harvested immediately after sacrifice and the skin was removed. Using a razor blade, the distal tip of the tail was removed to leave at least four functional spinal units, containing the two injured discs and two control discs. Further preparation was adapted from other reports of cryostat sectioning of soft tissue for AFM analysis of ECM.^7^, ^17^ The samples were embedded in OCT solution and frozen at −80°C. 10 μm thick sagittal sections were made using a Leica cryostat and cryostat tape to preserve the integrity of each section during cutting. The tape side of the section was adhered to a glass slide with a thin layer of cyanoacrylate glue applied using a brush. Slides were stored at 4°C.

#### 2.3.2 Image acquisition

**FIGURE 2:**
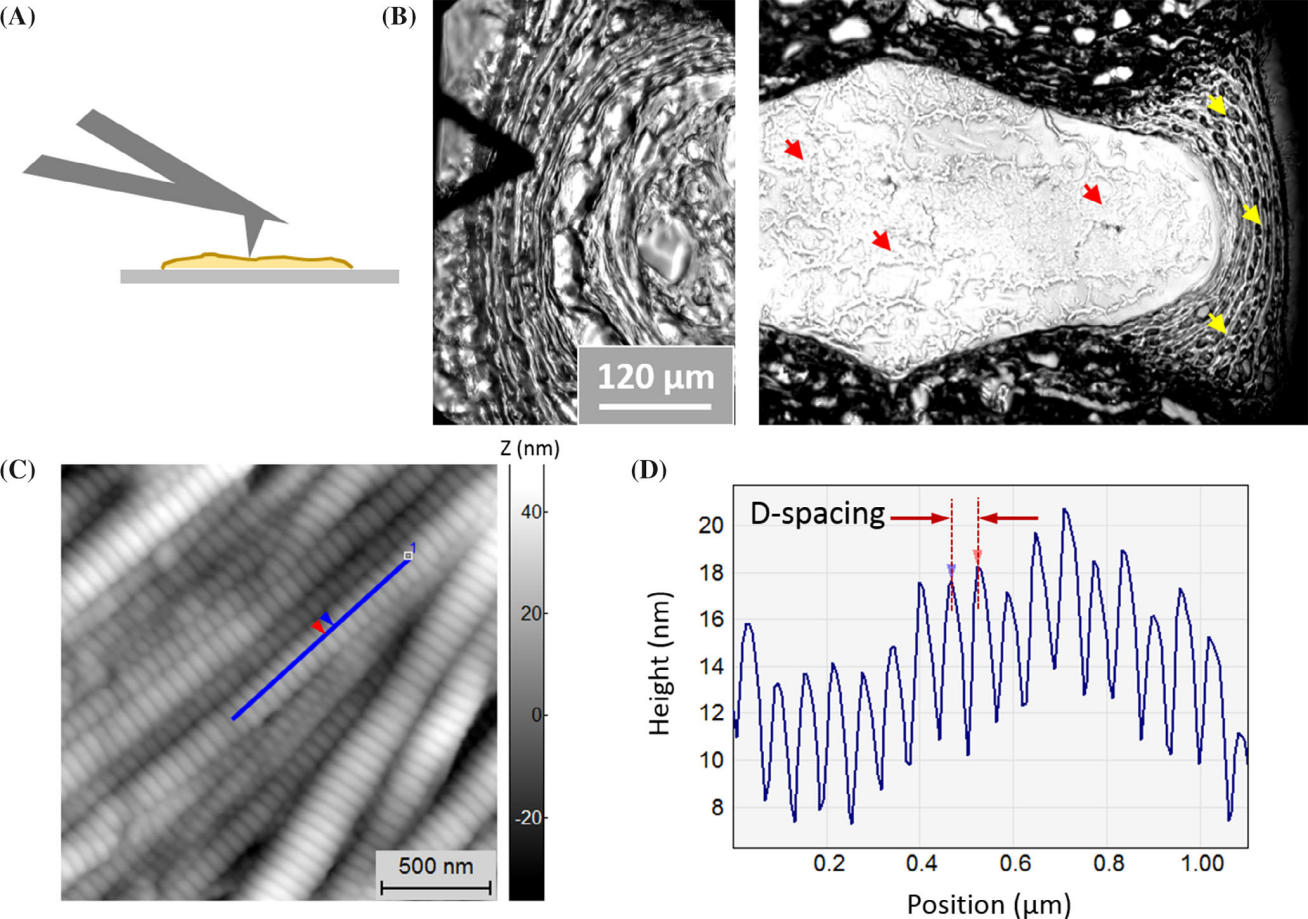
Data Acquisition. A, AFM images were acquired in contact mode. B, Imaging locations were selected using an inverted optical microscope. The imaging tip was positioned at locations distributed throughout the NP (red arrowheads) and the AF (yellow arrowheads). C, Representative topography images of collagen fibrils in the AF of IVD shown in B. Collagen fibrils are identified by the characteristic three‐dimensional banding pattern corresponding to D‐spacing. D, A line scan displaying the height profile of the fibril identified in C. The peak‐to‐peak distance is defined as the fibril D‐spacing. AF, annulus fibrosis; AFM, atomic force microscopy; IVD, intervertebral disc; NP, nucleus pulposus

All AFM imaging was carried out on 10 μm thick mid‐sagittal sections of tissue in air at room temperature. No dehydration procedures were performed. Imaging was conducted using an MFP‐3D‐Bio AFM (Asylum Research, Santa Barbara, CA) in contact mode with Bruker SNL‐10 probes (Bruker AFM Probes, Camarillo, CA; cantilever C, pyramid silicon nitride tip with nominal tip radius 2 nm, cantilever spring constant 0.24 N/m, resonance frequency 56 kHz). Line scan rates were set to 2 Hz or lower at 512 lines per frame. Initial scan sizes were either 30 or 20 μm. From these scans, imaging regions of interest were selected for further imaging at smaller scan sizes. Regions of interest were defined as areas with collagen fibrils aligned in sample cutting plane and any other areas with topographical details smaller than 10 μm. 10 μm and 3.5 μm images were obtained at each imaging region of interest to achieve high resolution of single collagen fibrils and other hierarchical structures. Samples were imaged with an equilibrium moisture content to that of the atmosphere in order to achieve optimal imaging resolution. An inverted microscope (Zeiss Axiovert A1, Oberkochen, Germany) was used in conjunction with the AFM to aid in selection of imaging locations (Figure 2B). A minimum of three locations were imaged from each region (AF and NP) for all IVDs imaged. The tissue region between AF and NP was imaged for sections containing a distinct transition zone. Requirements guiding image location selection include selecting r once per discTotal image counts used for quantitative analysis were as follows: 84 images in the AF distributed across 21 locations (four nondegenerate, five mild, five moderate, and seven severe), 31 images in the transition zone distributed across six locations (0 nondegenerate, two mild, three moderate, and one severe), and 55 images in the NP distributed across 13 locations (two nondegenerate, two mild, four moderate, and five severe). There were fewer images from transition regions because this region does not exist for nondegenerate or severely degenerate discs.

#### 2.3.3 Image processing and analysis

**FIGURE 3:**
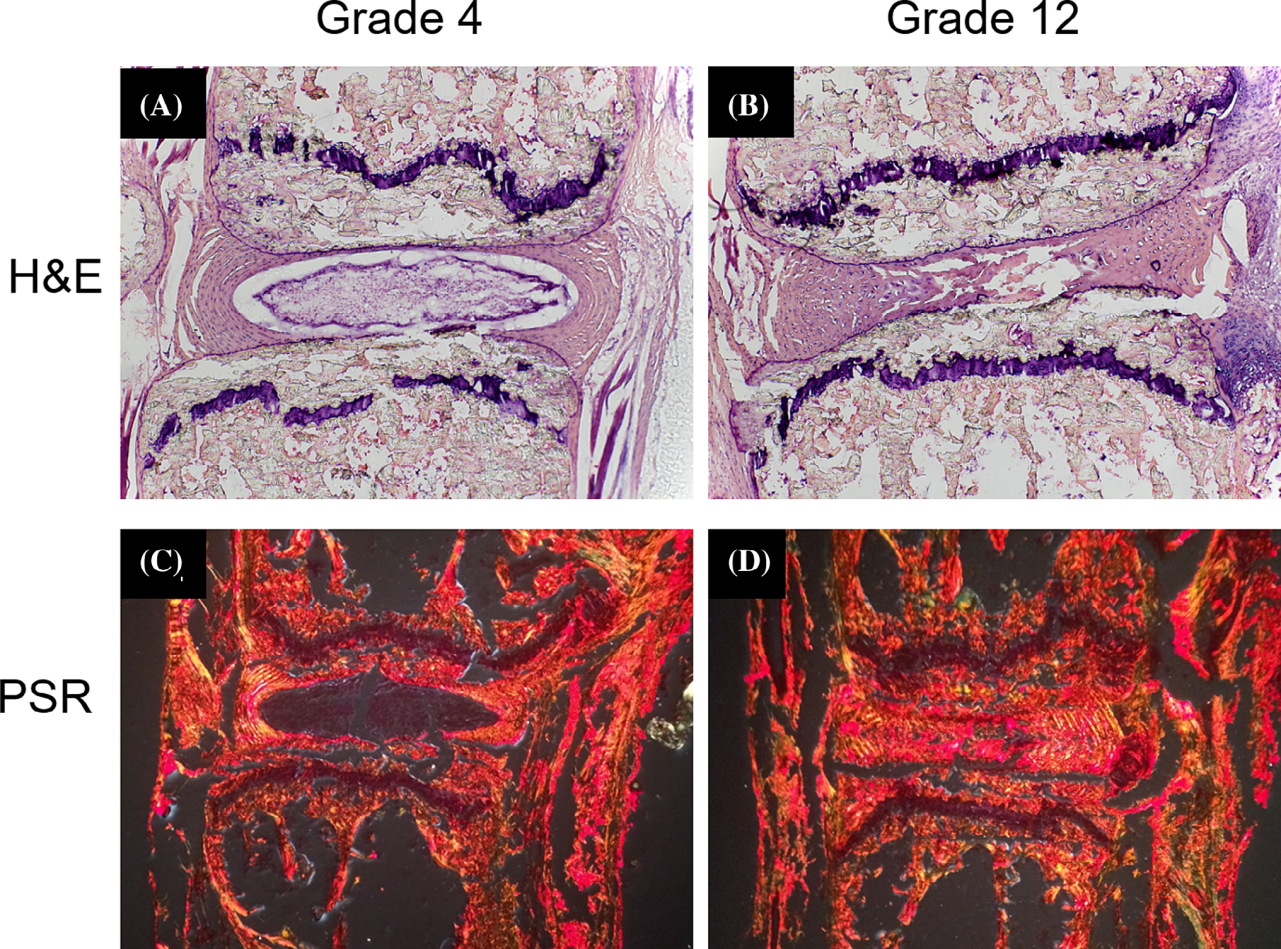
Injury‐induced degeneration of the IVD. Hematoxylin and Eosin (H&E) staining demonstrates the change in overall tissue architecture in a nondegenerate grade 4 disc (A, vs a severely degenerate disc grade 12, B. Picrosirius Red staining under polarized light shows the transition from a NP without collagen in a nondegenerate disc C, to a collagen‐rich and fibrotic NP in a severely degenerate disc, D. Degeneration in discs B, and D, was induced via injury. Discs A, and C, were intact controls. IVD, intervertebral disc; NP, nucleus pulposus

Gwyddion, an open source software for scanning probe microscopy, was used for image processing and data analysis. Throughout the analysis, topographic images were used with a plane subtraction and deflection images were unprocessed. Collagen fibrils were identified based on fibril topography (Figure 3C,D). Fibrils with a well resolved D‐spacing, or fibrils oriented parallel to the cutting surface of the sample, were included in the analysis for D‐spacing. The D‐spacing of individual collagen fibrils was measured from deflection AFM images using 2D Fast Fourier transforms using the SPIP software (Image Metrology, Horsholm, Denmark)^3^ (Supplemental Figure S1). Deflection images were used for analysis to measure fibril morphology independent of overall sample topography. Distributions of D‐spacing were plotted as both histograms and cumulative density functions (CDFs). The distributions were compared using Kolmogorov‐Smirnov statistical tests.

A novel microstructural feature we call *collagen toroids* (Figure 6) were identified and quantified using Gwyddion software. For a feature of interest to be defined as a *collagen toroid*, the topographical line scan must produce a peak around the rim of the feature with a depression in the middle. Additionally, evidence of collagen fibrils near (within a 3.5 × 3.5 μm scan) the feature must be present upon the zoomed in scan to confirm the feature was composed of ECM as opposed to an imaging artifact (Supplemental Figure S3). Quantification of the *collagen toroids* was based on aspect ratio measurements for the size of the feature (Supplemental Figure S2).

## 3 RESULTS

### 3.1 Histological evaluation of collagen and proteoglycan

Reproducible degeneration was found throughout the study period, in varying degrees of severity, as graded by the Masuda scale based on histologic sections (Figure 3).^14^ At 6 weeks postsurgery, noninjured discs located adjacent to injured discs showed signs of degeneration ranging from mild to severe. Discs were grouped based on their scores into nondegenerate (4), mild (grades 5‐6), moderate (7–9), and severe (10–12) degeneration. In the 0‐week postsurgery animals, 15 discs were graded a 4 (nondegenerate) and one disc was graded a 5 (mild). In the 2 week postsurgery group, 18 intact discs were graded a 4 (nondegenerate) and 18 injured discs were graded a range of scores from 5 (mild) to 11 (severe). In the 4 weeks postsurgery group, 18 intact discs were graded a 4 (nondegenerate) or 5 (mild). Eighteen injured discs in this group were graded with a range from 7 (moderate) to 12 (severe). In the 6 weeks postsurgery group, 12 intact discs were graded with a range from 4 (nondegenerate) to 10 (severe). Twelve injured discs in this group were graded with a range from 8 (moderate) to 11 (severe). The decision to categorize discs based on histological grade provided a systematic way to test our hypothesis and investigate ECM structure as a function of the amount of degeneration. All results are presented based on the stage of degeneration. The tissue level changes due to injury are summarized in Figure 3. In intact control discs (3A and 3C), there are clear boundaries between the NP and AF. Upon injury (3B and 3D), degenerative changes are observed. These include a merging of the NP and AF regions and reduction in disc height. Staining with PicroSirius red (Figure 3) clearly demonstrates the change in collagen distribution throughout the disc in an intact vs injured disc. In the intact, or nondegenerate disc, collagen is seen in the AF and not in the NP. In the severely degenerated disc, there is no NP border and collagen signal is detected with the stain throughout the entire disc.

### 3.2 Collagen D‐spacing distribution

**FIGURE 4:**
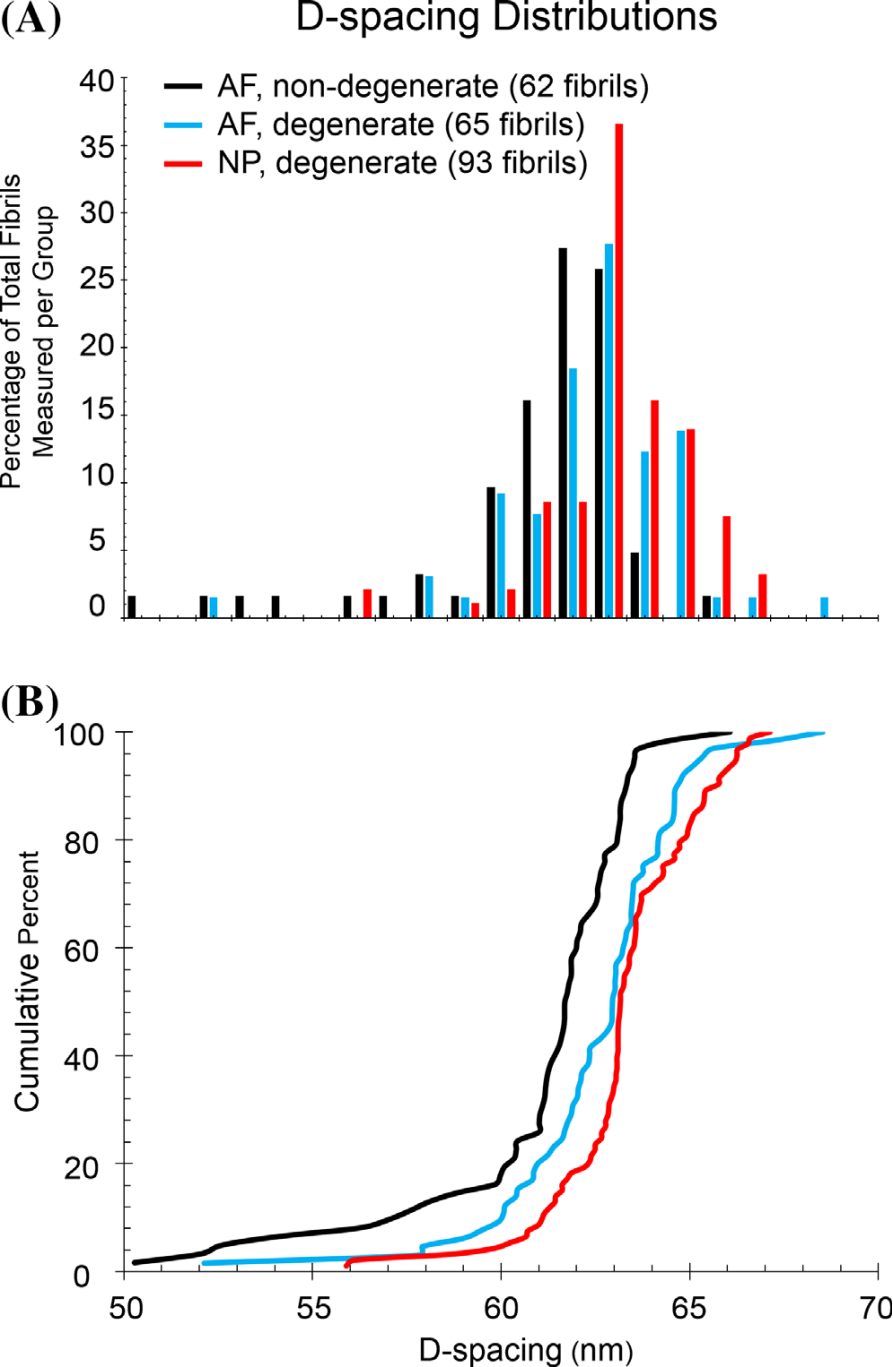
Histograms and cumulative density functions (CDFs) were used to compare distributions of D‐spacing based on region and grade of degeneration. The average values were not different from one another (61.1 ± 2.9, 62.6 ± 2.3, and 63.3 ± 1.9 nm for AF nondegenerate, AF degenerate, and NP degenerate, respectively). Distributions were compared using CDFs and Kolmogorov–Smirnov (KS) test statistics. The AF nondegenerate distribution was different from both of the degenerate distributions with statistical significance (*P* < .05). The AF and NP degenerate distributions were not different from one another (*P* > .05). AF, annulus fibrosis; NP, nucleus pulposus

**FIGURE 5:**
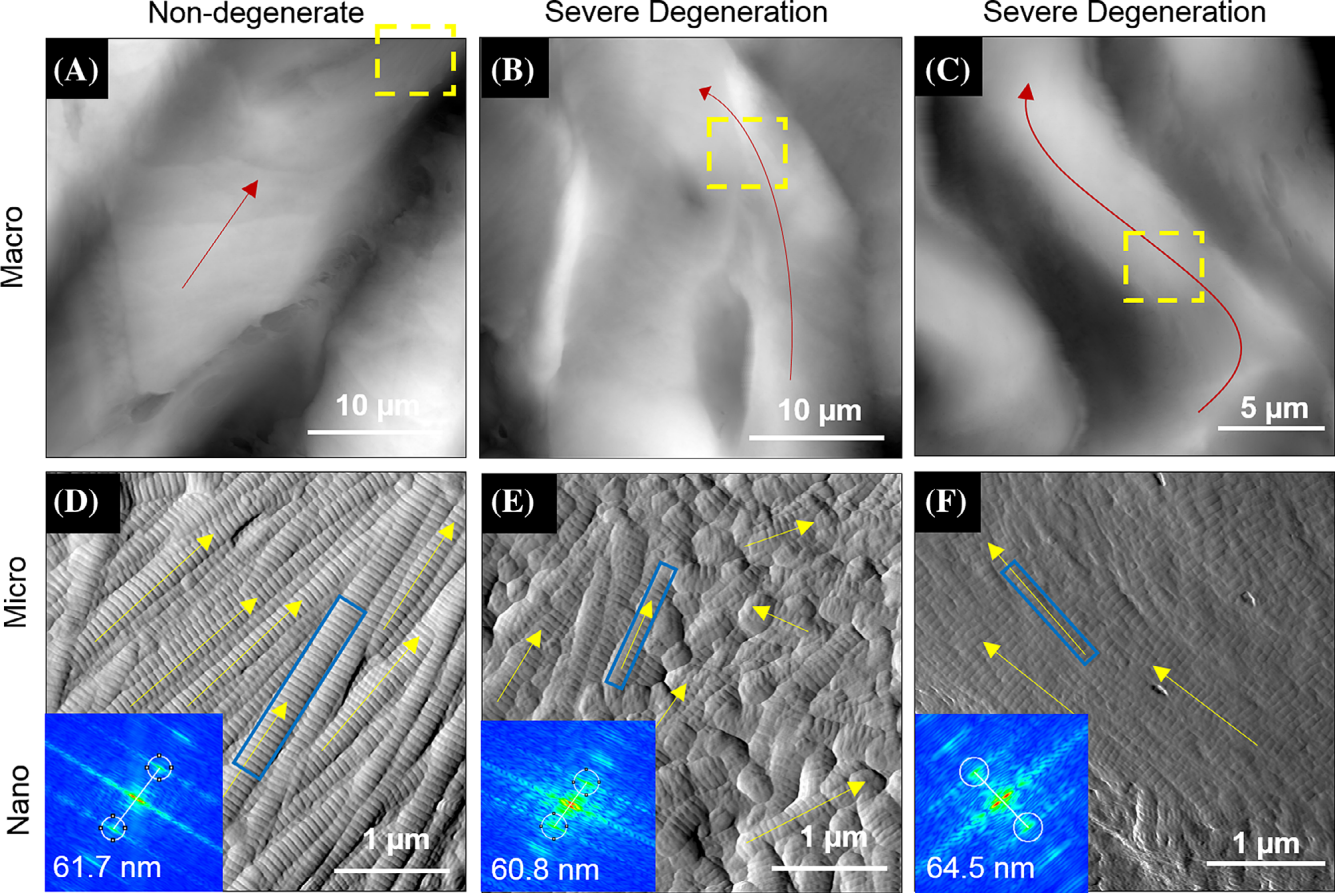
Hierarchical Structure of the annulus fibrosus during degeneration. Topographic large scans of the AF used to identify imaging regions of interest revealed lamellar structure consistent with histological observations of lamellar structure. Imaging regions are comparable to the tip location shown in Figure 2B. The red arrows indicate the axial direction of the lamellae for a representative nondegenerate case A, and two degenerate cases (B, and C,). Zoomed in 3.5 μm scans of the regions indicated with yellow boxes in A, B, and C, produce the deflection images in D, E, and F, respectively. In D and F, fibril alignment is oriented in the direction of the overall lamellar axis. In E, there is no overall collagen alignment direction. The inset images in D, E, and F, are two dimensional fast fourier transforms (FFTs) of the fibrils identified with a blue box. These FFTs were used to measure the D‐spacing of the indicated fibrils

Collagen fibril D‐spacing was used as a way measure nanoscale structure. The D‐spacing was measured from AFM deflection images and the distributions of these values were evaluated based on region (AF vs NP) and stage of degeneration. A total of 220 individual fibrils were measured across all stages of degeneration. The average values for D‐spacing were as follows: 61.1 ± 2.9 nm in nondegenerate AF, 62.6 ± 2.3 nm in degenerate AF, and 63.3 ± 1.9 nm in degenerate NP. There were not enough well‐defined fibrils in the nondegenerate NP to measure a D‐spacing distribution. Histograms and CDF are displayed in Figure 4. Kolmogorov‐Smirnov (KS) statistical tests were used to compare the distributions of D‐spacing in the CDFs. Fibrils measured from the AF of severely degenerated discs had a distribution of D‐spacing values shifted towards larger values than fibrils measured from AF of nondegenerate discs (*P* = .001). Additionally, fibrils measured from severely degenerated NP were shifted towards larger values compared to nondegenerate AF (*P* < .001). There was no statistical difference between the distributions of D‐spacing in fibrils measured from AF degenerated or NP degenerated discs. Representative images of collagen fibrils from each of these regions can be found in Figures 5D (nondegenerate AF), 5E and 5F (degenerate AF), and supplemental Figure S4 (degenerate NP).

### 3.3 Microstructural assessments

#### 3.3.1 Annulus fibrosus

In nondegenerate IVDs evaluated with AFM imaging the type I collagen fibrils comprising a lamella in the AF were aligned along the axis of that lamellae and formed large bundles that were approximately 5 to8 μm in width (Figure 5A,D). In IVDs with some degree of degeneration, the AF lamellar structure was disrupted and displayed a serpentine shape, consistent with histological criteria used to grade the IVD using the Masuda grading scale for degeneration. Collagen fibril alignment was occasionally disrupted, as seen in Figure 5. The topographic images in Figure 5B,C are both from severely degenerate AF and the lamellar structure is serpentine in both cases. The zoomed in deflection image for 5B reveals fibrils with no dominant fibril alignment direction (5E). In contrast, the zoomed in image of 5C reveals fibrils with preserved alignment along the axis of the larger lamellae (5F). No correlation was observed between the occurrence of this alignment disruption and stage of degeneration, weeks post injury, or imaging location in the AF.

#### 3.3.2 Nucleus pulopsus

**FIGURE 6:**
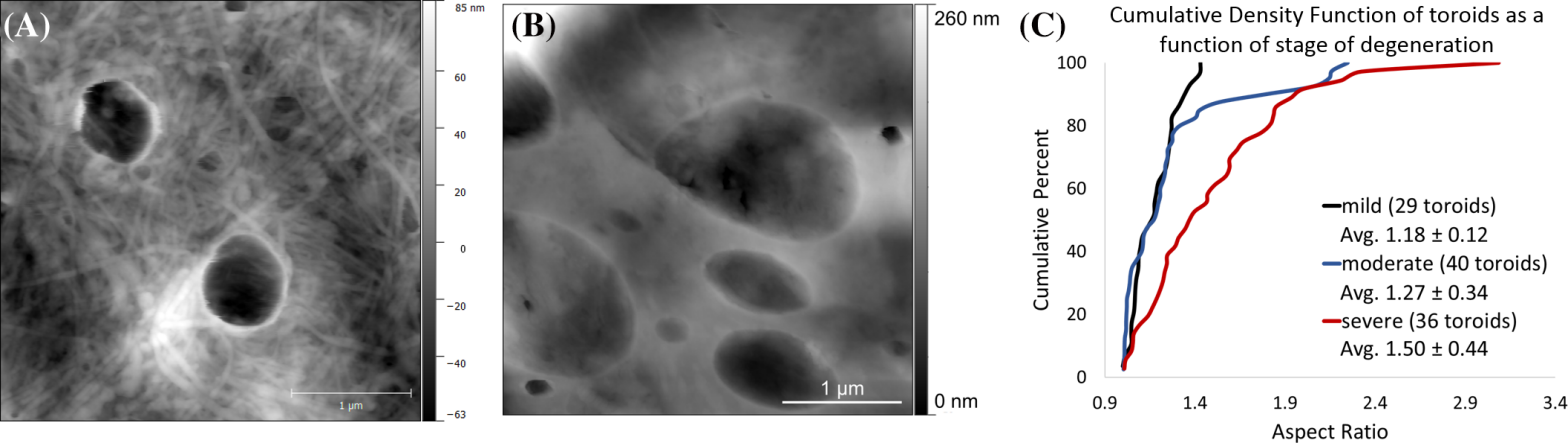
Representative topography AFM images of NP at progressive stages of degeneration. A, NP tissue from a mildly degenerate (grade 5) disc with circularly shaped collagen toroids. Fibrils of the extracellular matrix are resolved surrounding and within the structures. B, NP tissue from a severely degenerate (grade 11) disc with an increase in the number, size, and aspect ratio of toroids. C, Cumulative density function representing the aspect ratio of individual toroids: The aspect ratios of toroids in moderately degenerated disc (Masuda grades 7‐9) were distributed with an average of 1.13 ± 0.16. Severely degenerated disc (Masuda grades 10‐12) were significantly shifted towards higher aspect ratios (*P* < .005, Kolmogorov‐ Smirnov test) with an average of 1.50 ± 0.44. AFM, atomic force microscopy; NP, nucleus pulposus

A circular or oval shaped depression enclosed by ECM fibrils was consistently observed in the NP regardless of the grade of degeneration. This is a novel structural feature in tissue that we are calling *collagen toroids* (Figure 6). We chose to focus our characterization of toroids on the shape of the feature instead of overall size because shape is likely less sensitive to the sectioning plane being imaged. As the grade of NP degeneration increases, the shape becomes more oval. In mild or moderate grades of degeneration, the toroids are circular and have an aspect ratio of 1.12 ± 0.15. In severe grades of degeneration, the aspect ratios are 1.50 ± 0.44. The distribution of aspect ratios is significantly different (*P* < .005, Kolmogorov‐Smirnov test) between moderate and severely degenerated samples with the severely degenerated samples being skewed towards higher aspect ratios. Additionally, there were more toroids found in the NP than in other regions. 5.1% of images taken from the AF contained toroids (78 images total), 12.9% of images taken from the transition region had toroids (31 images total), and 32.7% of images from the NP contained toroids (55 images total). There were very few toroids found in the annulus (eight measurements) compared to the nucleus (69 measurements) and transition region (28 measurements).

## 4 DISCUSSION

Imaging with AFM provides unique advantages for investigating collagen structure in tissue.^18^ In contrast to other methods, tissue is unfixed, unstained, and measured directly with nanometer lateral resolution, making it an ideal technique to study ECM in the context of disease.^19^ AFM has been utilized to characterize type I collagen structure in skin,^7^ tendon^5^, ^7^ and bone.^5^ The findings based on these AFM studies have provided insights into disease that other techniques would not have provided.^4^, ^6^, ^8^, ^20^ The IVD is a collagen‐based tissue that remains under‐represented in this field of study. The only AFM images of IVD in the literature were not part of a systematic and quantitative characterization of ECM in the disc. An AFM image of disc was included in a methods paper^21^ to describe preparing tissue cryosections for AFM imaging. The only other AFM study providing imaging data focused on nanoindentation measurements and did not quantify imaging data.^22^ AFM studies conducted on articular cartilage have focused on nanoindentation and the effects of proteoglycan depletion on tissue stiffness as opposed to the imaging modality of AFM. Of the studies that have been conducted on articular cartilage, it has been concluded that tissue biomechanics can be largely impacted by ECM microstructure independent of composition.^23^ Articular cartilage is distinct from disc tissue with differences in structure, composition, and cell type. To our knowledge, a thorough study of ECM structure in the IVD has not been reported in the literature. Future studies will include nanoindentation to determine the impact of the findings we report here on the IVD biomechanics.

It is unknown if the structural changes we report can be generalized to human disc degeneration. However, collagen is a highly conserved across species, suggesting that our findings are likely to be translated to human IVD degeneration. Other limitations of the study include variability in the rate of degeneration in the surgical models and differences in of the biomechanical function of IVDs in humans and mice.

### 4.1 D‐spacing

The D‐spacing of type I collagen has been reported as 67 nm since the proposal of the Hodge‐Petruska model in 1964.^9^ Within the past decade, D‐spacing analyses on a fibril‐by‐fibril basis has demonstrated that this nanoscale feature of the collagen fibril exists as a distribution of values in skin, tendon, dentin, and bone.^5^, ^7^ In bone, this distribution is altered in response to disease states such as estrogen depletion and osteogenesis imperfecta.^24^ To date, there have not been significant differences observed in the average D‐spacing value as a function of tissue type, estrogen depletion, or drug treatment.^4^, ^5^, ^6^, ^7^, ^8^, ^20^, ^25^ Estrogen depletion has been shown to alter the distribution of D‐spacing values^6^ and drug treatment has been shown to alter the amount of animal‐to‐animal variation of D‐spacing in bundle fibrils in cortical bone.^20^ The D‐spacing data reported in this study for the IVD is the first time that a collagen‐based tissue has produced a distribution with a different average value and a different distribution in the nondisease state. The average values for D‐spacing in the IVD range from 61 to 63 nm (depending on region/degeneration) with the classical 67 nm value being at the higher end of the distribution. This is shifted towards lower values than all other collagen‐based tissues. The IVD is compositionally unique compared to skin, tendon, bone, and dentin. It contains higher amounts of other types of collagens, including types II, III, and IX, in addition to high amounts of proteoglycans all of which may contribute to alterations in D‐spacing in the disc.

Degeneration of the disc resulted in the distribution of D‐spacing being shifted towards higher values (*P* < .05) with no change in the average value. The molecular mechanisms that give rise to collagen fibril D‐spacing and alterations in D‐spacing is still an unknown. We hypothesize the compositional changes during degeneration play a role in the altered fibril structure we have reported. Alteration in proteoglycan levels, both in type and quantity, is a known consequence of IVD degeneration. We hypothesize that this change in proteoglycan levels is involved in changing the D‐spacing distributions as varying proteoglycan levels is known to alter other features of the collagen fibril, such as diameter and uniformity.^26^ Additionally, estrogen depletion, which has been linked to change in proteoglycan levels, has been shown to shift the D‐spacing distribution towards lower values in ovine bone compared to sham animals.^6^ This shift in D‐spacing approached the values observed in degenerate disc, lending further credibility to our hypothesis. An additional source of compositional change is through the accumulation of advanced glycation end products (AGEs). Although we have not directly measured these in our samples, it is worth noting that AGEs have been reported in the degenerate disc^27^ and AGEs have been reported to reduced collagen D‐spacing for collagen fibrils deposited onto mica.^28^

The need for large datasets of individual D‐spacing fibril measurements is demonstrated in Figure 5. An example of a nanoscale measurement from each of three images is shown in 5D, 5E, and 5F. The three images are representative of aligned fibrils from the AF of nondegenerate disc (5D), fibrils with disrupted alignment from the AF of degenerate disc (5E), and aligned fibrils from the AF of degenerate disc (5F). The inset FFTs correspond to the blue outlined fibrils. The small variations in individual D‐spacings from these three measurements (61.7, 60.8, and 64.5 nm) do not suggest any disease related changes. These three measurements highlight the importance of measuring many fibrils from many locations. To detect disease related changes, D‐spacing distributions from many measurements (as reported for the nanostructure analysis) need to be analyzed.

### 4.2 Microstructure

The results regarding collagen alignment in this study are observational. It is important to report these observational findings because they inform the development of novel image analysis tools to quantify the current data set or future AFM data sets from IVD. A fibril alignment parameter (FAP) has been developed and used to quantify alterations to fibril alignment in bone as a function of estrogen depletion and drug treatment.^20^ Our imaging data set can be used by us or others to develop an analogous quantification method.

Toroidal structures formed from collagen molecules were first reported by Cooper in 1969 from electron microscopy studies of calf‐skin tropocollagen precipitated onto a substrate.^29^ To our knowledge, the current study is the first report of the collagen toroid in biological tissue. The structural feature we have called collagen toroids were routinely observed in the NP and not in the AF. The NP is a gelatinous structure with a high water content and relatively high amount of proteoglycan. The glycosaminoglycan to hydroxyproline, which is indicative of collagen, ratio is approximately 27:1 in the NP and 3:1 in other cartilaginous tissues in normal young adults.^30^ The AF is composed of highly organized type I collagen fibrils arranged in concentric lamellae. The compositional differences between these two structures are likely responsible for the differences in structures that are observed with AFM. We hypothesize that the collagen toroids are formed by type II collagen with the size and shape controlled by the associated proteoglycans. With progressive IVD degeneration, the ratio of glycosaminoglycan to hydroxyproline dramatically decreases.^30^ Studies have suggested than fibril diameter, length, and fibrillogenesis are controlled by proteoglycan content in tissue, particularly by a class of proteoglycans called small leucine‐rich proteoglycans (SLRPs).^31^, ^32^ During fibrillogenesis, mature fibrils are formed from early 1 to 3 μm early fibrils that fuse end‐to‐end. SLRPs coat the surfaces of fibrils to prevent these early fibrils from fusing together at the incorrect place.^33^ In older tissues, fibril fusion can occur tip‐to‐shaft in addition to tip‐to‐tip. This causes branching of collagen. Electron microscopy shows that the C‐terminal tips of unipolar fibrils fuse with the shafts of other fibrils to form these branches. Mice without SLRPs have more branching.^33^ These observations suggest that changing proteoglycan levels can alter fibril branching and tissue architecture at the fibril level. We hypothesize that the toroids (a change in tissue architecture) are related to changes in proteoglycan levels resulting from the degenerative process. Additional studies will need to be conducted to confirm this hypothesis.

The findings we report are not likely to be an artifact of sample preparation or imaging. It is important to emphasize that the IVD samples are not fully dehydrated even when AFM imaging is performed in air. To be fully dehydrated, the samples would need to be imaged in ultra‐high vacuum, as is done with electron microscopy. Imaging in air assumes a hydration layer on the surface of both the imaging tip and sample. The ability to image samples without full dehydration and without harsh preparation treatments is an advantage to using AFM for biological specimens. Our IVD samples were in a similar hydration state to the samples included in D‐spacing studies on dermis and tendon.^4^, ^5^, ^7^ When reporting the methodology for measuring D‐spacing using AFM images, Erickson et al^3^ performed an analysis of sheep dermis collagen fibrils. Twenty fibrils were imaged in air and in water. There was no correlation between the imaging environment and the small D‐spacing measurement changes, which averaged 0.9 nm, far smaller than the width of reported D‐spacing distributions. When considering the collagen toroids, the feature is always observed embedded into the tissue sample. Intact fibrils can be observed in and around the toroid structures. In 6A, fibrils can be observed curving towards and feeding into the structure. In supplemental Figure S3, we demonstrate the requirement for matrix around the perimeter of the structure as a definition for our *collagen toroid* structure. Additionally, all structures we identified as *toroids* were circular or oval in shape and topographically continuous with surrounding tissue. If these structures were a result of tears or contraction during sample preparation, those regions would likely not be flat enough to image with AFM and we do not expect to observe circles and ovals. For these reasons, the toroids are likely true architectural features of the IVD. Most importantly, all samples in the current study were prepared and imaged using identical protocols. Any differences reported are likely true degenerative‐related changes.

### 4.3 Significance and future directions

Based on X‐ray diffraction data, type I collagen was thought to have a single value for D‐spacing. It is only within the past decade that the field has recognized the intrinsic distribution of D‐spacing values and the importance of these distribution in relation to both disease and tissue architecture. These studies have focused on type I collagen fibrils in the tissues of bone, tendon, and skin. Although D‐spacing is a biologically significant nanoscale feature of type I collagen, there are many remaining questions regarding molecular mechanisms involved and implications for mechanical function of tissue. The addition of the IVD, and the degeneration mouse model, to this field of study opens doors to provide insight for these questions. The IVD is highly heterogeneous and compositionally unique from any other collagen‐based tissue, providing a unique perspective on collagen structure and function. Additionally, the ECM composition changes as a function of degeneration. This can be used to study how D‐spacing distributions change as a function of ECM composition.

Nanoscale structure of ECM, particularly D‐spacing distributions of type I collagen, is altered in response to both disease (estrogen depletion,^4^, ^6^, ^8^, ^20^ osteogenesis imperfecta^3^) and drug treatment^8^, ^20^ in a variety of species (ovine, rabbit, and monkey) and tissue types (bone, skin, tendon, and dentin). Although the direct impact on mechanical properties is not fully understood, nanoscale structural heterogeneity in tissue ECM is an important property of the tissue that can play a role in disease.^18^ These changes to D‐spacing distributions require measurements of individual fibrils and cannot be detected with techniques currently used in the field of spinal research. Application of AFM imaging to IVD allows for detection of structural heterogeneity at the micro‐ and nano‐ scales as well as the discovery of novel morphological features. We have reported methods and the first set of AFM‐based imaging data for nanoscale and microscale structure of ECM in IVD. Our data is unique with the field of IVD research field and provides details of structural changes at the nanoscale and microscale levels. Additionally, we have reported the first observation of *collagen toroids* in tissue in the context of a disease state. Our methods can be extended to study structural details within the IVD of a wide variety of animal models and treatments studies. Future directions to enhance interpretation of our work include sub‐micron compositional analyses, quantification of fibril alignment, and expanding the imaging data set to other animal models and treatments. Long term goals of this field include determining the relationship(s) between ECM structural changes and disc mechanics.

## CONFLICT OF INTEREST

The authors declare no potential conflict of interest.

## AUTHOR CONTRIBUTIONS

Cauble contributed to AFM sample preparation, data collection and image analysis. Cauble, Mancini and Kalinowski contributed to histological assessments. Mancini and Kalinowski contributed to animal surgeries. Cauble, Lykotrafitis, and Moss contributed to study design, results interpretation, and writing the manuscript. All authors have read and approved submission.

## Supporting information


**Supplemental Figure S1** Measuring fibril D‐spacing with SPIP. A. An imaging region for collagen fibril Dspacing analysis was selected from 10 x 10 μm scans. From the deflection image shown in panel A, a smaller imaging area with visible collagen fibrils was selected for subsequent imaging. B. After obtaining a 3.5 x 3.5 μm scan, individual collagen fibrils were selected for analysis. A rectangular area of interest (AOI) was drawn around a single fibril in SPIP software. C. The FFT module in SPIP was used to measure the Dspacing of the individual fibril. Using this method, the D‐spacing measurement represents the average Dspacing value along the fibril enclosed within the AOI.

**Supplemental Figure S2** Defining and Identifying Collagen Toroids. We have identified collagen toroids as a ring‐like structural feature composed of extracellular matrix that is enclosing a topographical depression. For positive identification of a collagen toroid, evidence of extracellular matrix must be present both surrounding and within the feature. This would ensure the feature was not an imaging artifact. As an example, the feature identified with a red arrow in panel A was scanned at increasingly smaller scan sizes. In panel B, the yellow arrows indicate evidence of extracellular matrix in the tissue surrounding the feature. An additional scan was required to identify extracellular matrix within the feature (C). The blue arrow indicates collagen fibrils. The green arrows highlight the presence of fibrillar‐like structures with widths ranging from 60‐110 nm.

**Supplemental Figure S3** Aspect Ratio Measurements for Collagen Toroids. To quantify structural features identified as collagen toroids, we measured the length of the major and minor axis using line scans of the topographic scan (B). Peak‐to‐peak distance was measured and the aspect ratio was calculated (D). Examples of measurements from a mildly degenerate (A) vs severely degenerate (B) nucleus pulposus can be seen in C.

**Supplemental Figure S4** Representative image of collagen fibrils from the NP of a severely degenerate disc. The imaging location is shown in the inset image.
