# Supplementary figures and images for: Atomic force microscopy imaging for nanoscale and microscale assessments of extracellular matrix in intervertebral disc and degeneration
Source: JOR Spine. 2020 Sep 23;3(3):e1125. doi: 10.1002/jsp2.1125 (PMC7524250; doi:10.1002/jsp2.1125)

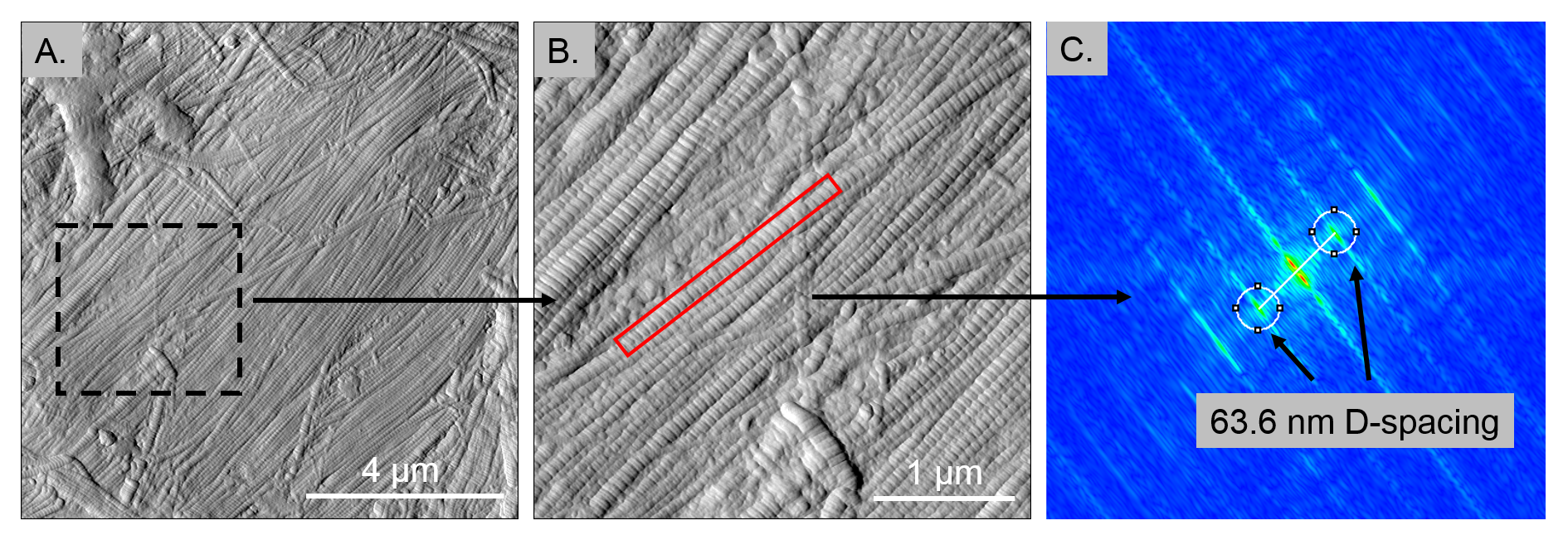

Supplement: Supplementary file 1 — Supplemental Figure S1 Measuring fibril D‐spacing with SPIP. A. An imaging region for collagen fibril Dspacing analysis was selected from 10 x 10 μm scans. From the deflection image shown in panel A, a smaller imaging area with visible collagen fibrils was selected for subsequent imaging. B. After obtaining a 3.5 x 3.5 μm scan, individual collagen fibrils were selected for analysis. A rectangular area of interest (AOI) was drawn around a single fibril in SPIP software. C. The FFT module in SPIP was used to measure the Dspacing of the individual fibril. Using this method, the D‐spacing measurement represents the average Dspacing value along the fibril enclosed within the AOI. [file JSP2-3-e1125-s001.png]

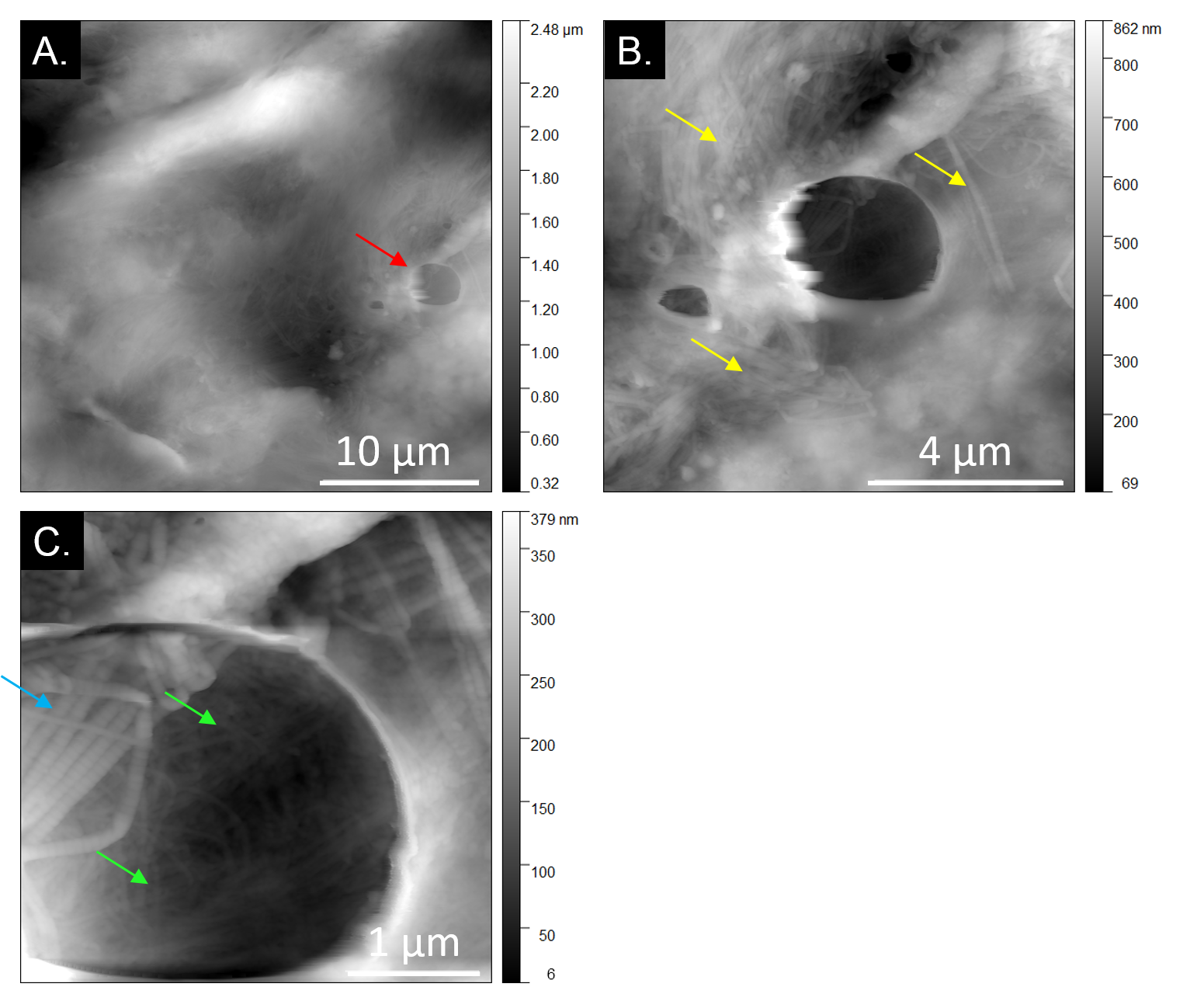

Supplement: Supplementary file 2 — Supplemental Figure S2 Defining and Identifying Collagen Toroids. We have identified collagen toroids as a ring‐like structural feature composed of extracellular matrix that is enclosing a topographical depression. For positive identification of a collagen toroid, evidence of extracellular matrix must be present both surrounding and within the feature. This would ensure the feature was not an imaging artifact. As an example, the feature identified with a red arrow in panel A was scanned at increasingly smaller scan sizes. In panel B, the yellow arrows indicate evidence of extracellular matrix in the tissue surrounding the feature. An additional scan was required to identify extracellular matrix within the feature (C). The blue arrow indicates collagen fibrils. The green arrows highlight the presence of fibrillar‐like structures with widths ranging from 60‐110 nm. [file JSP2-3-e1125-s002.png]

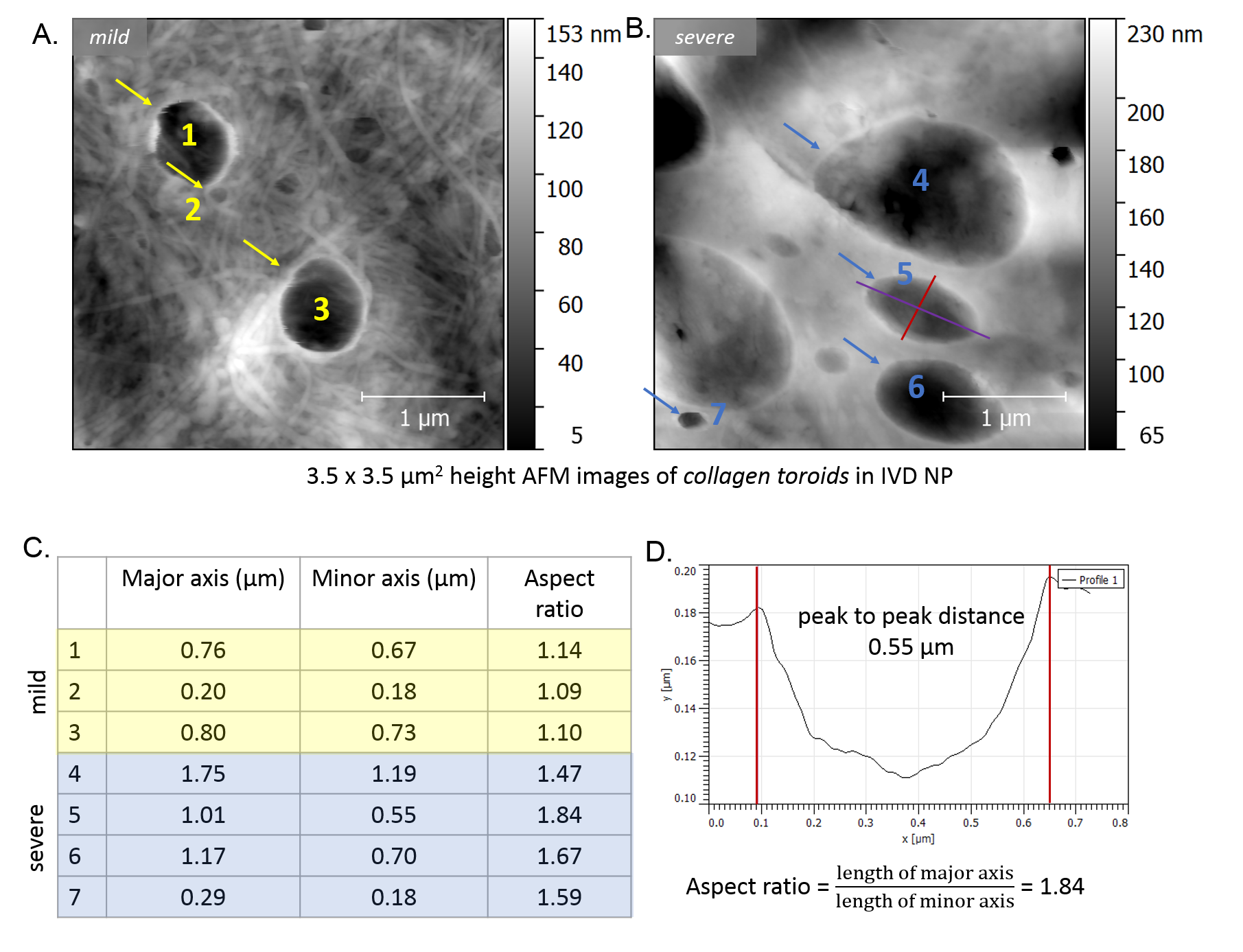

Supplement: Supplementary file 3 — Supplemental Figure S3 Aspect Ratio Measurements for Collagen Toroids. To quantify structural features identified as collagen toroids, we measured the length of the major and minor axis using line scans of the topographic scan (B). Peak‐to‐peak distance was measured and the aspect ratio was calculated (D). Examples of measurements from a mildly degenerate (A) vs severely degenerate (B) nucleus pulposus can be seen in C. [file JSP2-3-e1125-s003.png]

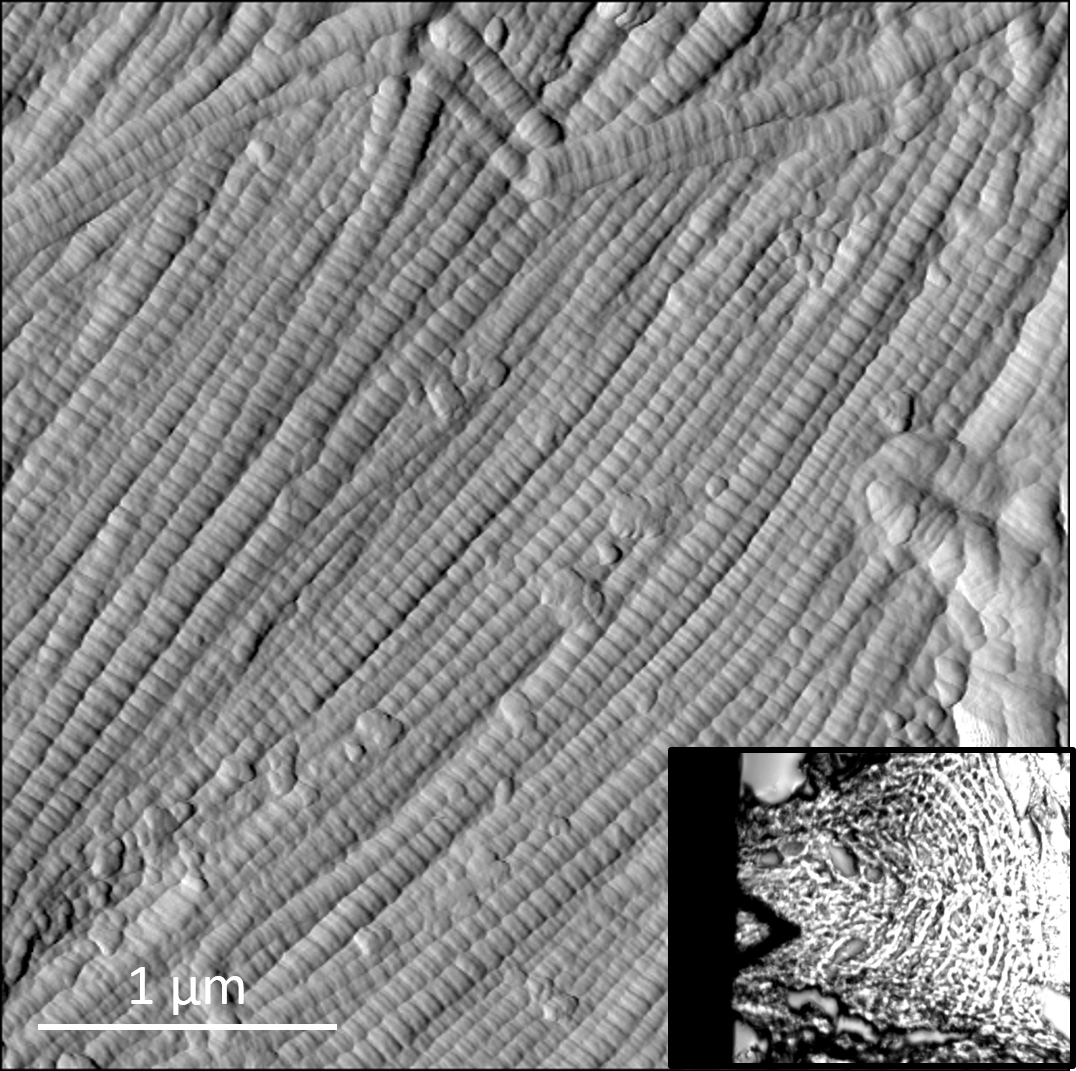

Supplement: Supplementary file 4 — Supplemental Figure S4 Representative image of collagen fibrils from the NP of a severely degenerate disc. The imaging location is shown in the inset image. [file JSP2-3-e1125-s004.png]
